# Epidemiology of multiple sclerosis: results from a large observational study in the UK

**DOI:** 10.1007/s00415-015-7796-2

**Published:** 2015-06-13

**Authors:** Susan S. Jick, L. Li, G. J. Falcone, Z. P. Vassilev, M.-A. Wallander

**Affiliations:** Boston Collaborative Drug Surveillance Program (BCDSP), Boston University School of Public Health, 11 Muzzey Street, Lexington, MA 02421 USA; Department of Neurology, Massachusetts General Hospital, Boston, MA USA; Bayer Healthcare Pharmaceuticals, Whippany, NJ USA; Department of Public Health and Caring Science, Uppsala University, Uppsala, SE Sweden

**Keywords:** Multiple sclerosis, Epidemiology, Cohort Analysis, Mortality

## Abstract

Multiple sclerosis (MS) progression to mortality may not be solely determined by the underlying autoimmune process. We conducted a study in a large cohort of MS patients with the aim of describing characteristics of MS patients and identification of predictors for all-cause mortality in this patient group. We performed a retrospective analysis of primary care data from the UK Clinical Practice Research Datalink. Incident MS cases diagnosed between 1993 and 2006 were identified and validated using electronic and original medical records. Patients were followed to identify deaths; hazard ratios (HRs) and 95 % confidence intervals (CIs) were estimated using Cox proportional regression with age as time-scale. In total, 1713 incident MS cases were identified. Following MS diagnosis, frequent comorbidities were infections (80 %), and depression (46 %). Adjusted HRs (95 % CIs) for all-cause mortality were: 2.0 (1.2–3.4) for current smoking; 7.6 (3.2–17.7) for alcohol abuse; 2.7 (1.6–4.5) for pneumonia and influenza; 4.1 (2.7–6.3) for urinary tract infections; 2.2 (1.2–4.2) for heart disease and 4.9 (2.9–8.0) for cancer. Our results suggest that MS survival is influenced not only by the underlying autoimmune process, but also by patient comorbidities and lifestyle factors.

**Electronic supplementary material:**

The online version of this article (doi:10.1007/s00415-015-7796-2) contains supplementary material, which is available to authorized users.

## Introduction

Multiple sclerosis (MS) is the most prevalent disabling neurological disease among young adults [1]. Despite recent advancements in the understanding of this condition, many aspects of its aetiological mechanisms remain poorly understood. Understanding the aetiology and clinical onset of MS is of increasing importance as evidence suggests an almost universal increase in the incidence and prevalence of the disease over the last five decades [1, 2]. An important question is whether risk factors and comorbidities for the disease interact with the underlying autoimmune process, or the drugs used to treat it, and the extent that this may influence mortality. We previously reported on the results of a study that compared risk factors for and rates of mortality in MS patients with non-MS patients using a large, validated UK primary care database [3]. The risk of mortality was found to be increased in MS compared to non-MS patients and MS patients had more comorbidities and co-medications than non-MS patients. Using the same database we now aim to describe predictors of all-cause mortality in MS patients only, to better understand the interaction between the autoimmune process and comorbidities. We hypothesised that factors other than the underlying autoimmune process play an important role in determining survival in MS patients.

## Methods/patients

### Study design

We conducted a population-based observational study using data from the UK’s Clinical Practice Research Datalink (CPRD), formerly the General Practice Research Database (GPRD). The CPRD contains prospectively collected data on all aspects of medical care for around 8 million people in the UK. Validation studies have shown the quality of the CPRD data, which have been used for several MS studies [3–10], to be generally high [11–14]. Also available are paper records that describe referrals, hospitalizations and other relevant medical history, often covering the patient from a young age. These provide an ideal resource to validate MS diagnoses and determine dates of MS onset. Details of the database have been described elsewhere [11–14]. This study was reviewed and approved by the Independent Scientific Advisory Committee for UK Medicines and Healthcare products Regulatory Agency database research.

### Case definition

We identified all patients in the CPRD who had a first diagnosis code (Online Resource 1) for MS between 1st January 2001 and 31st December 2006 who had at least 2 years of information in their electronic records. Cases were identified only through 2006 to provide sufficient follow-up to develop new outcomes, since the primary objective of this research was to study the prognosis of people with MS. Among all potential cases we classified each according to the likelihood that the MS diagnosis was valid according to the 2005 revisions to the McDonald MS criteria where data were available [3, 15]. This process resulted in a series of cases validated through detailed paper and electronic records, or electronic records only. For patients with paper records, we were able to get additional information on the type of MS [relapsing–remitting MS (RRMS), primary progressive MS (PPMS), secondary progressive MS (SPMS), and unknown type] and the presence of MS symptoms at onset. We classified symptoms, where available, into four main groups: sensory, motor, optic neuritis, and other symptoms (including other optic and visual anomalies, other cranial nerve anomalies, dysarthria, and asthenia). For those with no symptoms recorded, we treated them as a separate group with missing symptoms. In addition, all incident and prevalent MS cases still present in the 2012 data update, identified and validated in our earlier studies conducted using the GPRD (1993 and 2000) [5–10] were added to this 2001–2006 case set. Further details of the study design and validation process have been published previously [3].

### Covariates

Information on the following covariates was extracted from the database: age at first MS diagnosis, sex, and lifestyle factors closest to and before the first MS diagnosis including smoking, body mass index and alcohol abuse (Table 1). Information on acute infections (recorded on or after first MS diagnosis), chronic comorbidities (recorded at any time in the database), and MS treatment (recorded on or after first MS diagnosis) were also extracted. Comorbidities and MS treatment evaluated are listed in Table 2. MS treatment included disease-modifying medications (interferons, glatiramer acetate, mitoxantrone, and natalizumab), steroids, and symptomatic management drugs (tizanidine, baclofen, amantadine HCL, modafinil, and fampridine). We were unable to obtain complete information on use of disease-modifying medications because, in the UK, they are generally prescribed in secondary care and are not always captured in general practice (GP) records.

**Table 1:** Basic characteristics of incident MS cases at MS diagnosis, stratified by status: alive/dead

	Alive *N* = 1598 *n* (%)	Dead *N* = 115 *n* (%)	Overall *N* = 1713 *n* (%)
Mean age in years (SD)	41.1 (11.2)	51.4 (14.5)	41.8 (11.7)
Year of birth			
<1940	55 (3.4)	32 (27.8)	87 (5.1)
1940–1949	252 (15.8)	30 (26.1)	282 (16.5)
1950–1959	423 (26.5)	26 (22.6)	449 (26.2)
1960–1969	493 (30.9)	21 (18.3)	514 (30.0)
1970+	375 (23.5)	6 (5.2)	381 (22.2)
Sex			
Female	1181 (73.9)	79 (68.7)	1260 (73.6)
Male	417 (26.1)	36 (31.3)	453 (26.4)
Smoking status			
Never	715 (44.7)	31 (27.0)	746 (43.6)
Current	501 (31.4)	42 (36.5)	543 (31.7)
Former	187 (11.7)	21 (18.3)	208 (12.1)
Unknown	195 (12.2)	21 (18.3)	216 (12.6)
BMI (kg/m^2^)			
<18.5	38 (2.4)	4 (3.5)	42 (2.5)
18.5–24.99	615 (38.5)	47 (40.9)	662 (38.7)
25.0–29.99	372 (23.3)	26 (22.6)	398 (23.2)
≥30	229 (14.3)	13 (11.3)	242 (14.1)
Unknown	344 (21.5)	25 (21.7)	369 (21.5)
Alcohol abuse	17 (1.1)	8 (7.0)	25 (1.5)
Type of MS^a^			
RRMS	631 (78.7)	24 (49.0)	655 (77.0)
PPMS	108 (13.5)	10 (20.4)	118 (13.9)
SPMS	49 (6.1)	10 (20.4)	59 (6.9)
Unknown	14 (1.7)	5 (10.2)	19 (2.2)
Symptoms at onset^a^			
Sensory	241 (30.0)	6 (12.2)	247 (29.0)
Motor	186 (23.2)	15 (30.6)	201 (23.6)
Optic neuritis	164 (20.4)	7 (14.3)	171 (20.1)
Multiple symptoms^b^	42 (5.2)	7 (14.3)	49 (5.8)
Other	98 (12.2)	5 (10.2)	103 (12.1)
Missing	71 (8.9)	9 (18.4)	80 (9.4)

*BMI* body mass index, *MS* multiple sclerosis, *PPMS* primary progressive MS, *RRMS* relapsing-remitting MS, *SD* standard deviation

^a^Among 851 MS cases confirmed by original records (1993–2006)

^b^49 MS patients had multiple symptoms at onset

**Table 2:** Comorbidities and MS medications among incident MS cases, stratified by status: alive/dead

	Alive *N* = 1598 *n* (%)	Dead *N* = 115 *n* (%)	Overall *N* = 1713 *n* (%)
Acute infections recorded on or after first MS diagnosis			
Pneumonia and influenza	112 (7.0)	29 (25.2)	141 (8.2)
Other acute respiratory infection	751 (47.0)	53 (46.1)	804 (46.9)
Urinary tract infection	495 (31.0)	60 (52.2)	555 (32.4)
Other infection	1063 (66.5)	62 (53.9)	1125 (65.7)
Chronic comorbidities recorded at any time in the database			
COPD and asthma	315 (19.7)	28 (24.3)	343 (20.0)
Depression	730 (45.7)	55 (47.8)	785 (45.8)
Diabetes	79 (4.9)	14 (12.2)	93 (5.4)
Hypertension	232 (14.5)	21 (18.3)	253 (14.8)
Heart disease^a^	48 (3.0)	18 (15.7)	66 (3.9)
Cancer	83 (5.2)	26 (22.6)	109 (6.4)
Co-medications recorded on or after first MS diagnosis			
MS treatment^b^	1028 (64.3)	85 (73.9)	1113 (65.0)

*COPD* chronic obstructive pulmonary disease, *MS* multiple sclerosis

^a^Includes ischemic heart disease, angina, myocardial infarction, and heart failure

^b^Includes disease-modifying medications (interferons, glatiramer acetate, mitoxantrone, and natalizumab), steroids, and symptomatic management drugs (tizanidine, baclofen, amantadine HCL, modafinil, and fampridine)

### Statistical analysis

Patient characteristics were described and predictive factors for all-cause mortality were first identified by calculating crude hazard ratios (HRs) with 95 % confidence intervals (CIs) using univariate Cox proportional regression analyses. Because death was the outcome of interest, information on acute infections closest to the time of death was captured. We identified the last instances of each of the acute infections before end of follow-up and used that event date to divide follow-up time into two periods: one not exposed to acute infection and one exposed to acute infection. If chronic comorbidities were recorded on or before the first MS diagnosis, follow-up time was treated as exposed to chronic comorbidities. If chronic comorbidities developed during follow-up, we used the first event date to divide follow-up time into two periods: one not exposed to chronic comorbidities and one exposed to chronic comorbidities. MS treatment was treated as time-varying in the Cox regression models where total follow-up time was divided into one-year periods and we identified whether these medications were prescribed at least once in each period. Given that age is a known risk factor for mortality and also an important confounder of the associations between the studied chronic comorbidities (such as heart disease, cancer, etc.) and mortality, we used age as the time-scale in the Cox regression models, where patients entered the analysis at the age at first diagnosis of MS and exited at their death or censoring age, whichever was earlier. We also estimated the adjusted HRs and 95 % CIs for each predictive factor in the Cox proportional models which included all predictive factors as well as year of birth. Because the proportional hazards assumption may not be met across age, we divided the data into one-year age strata for each MS patient and fit models separately within each age stratum. Since age is the time scale, these age strata refer to attained age, rather than to age at cohort entry. All analyses were conducted using SAS version 9.3 (SAS Institute, Cary, NC).

## Results

### Case validation and selection

**Fig. 1:**
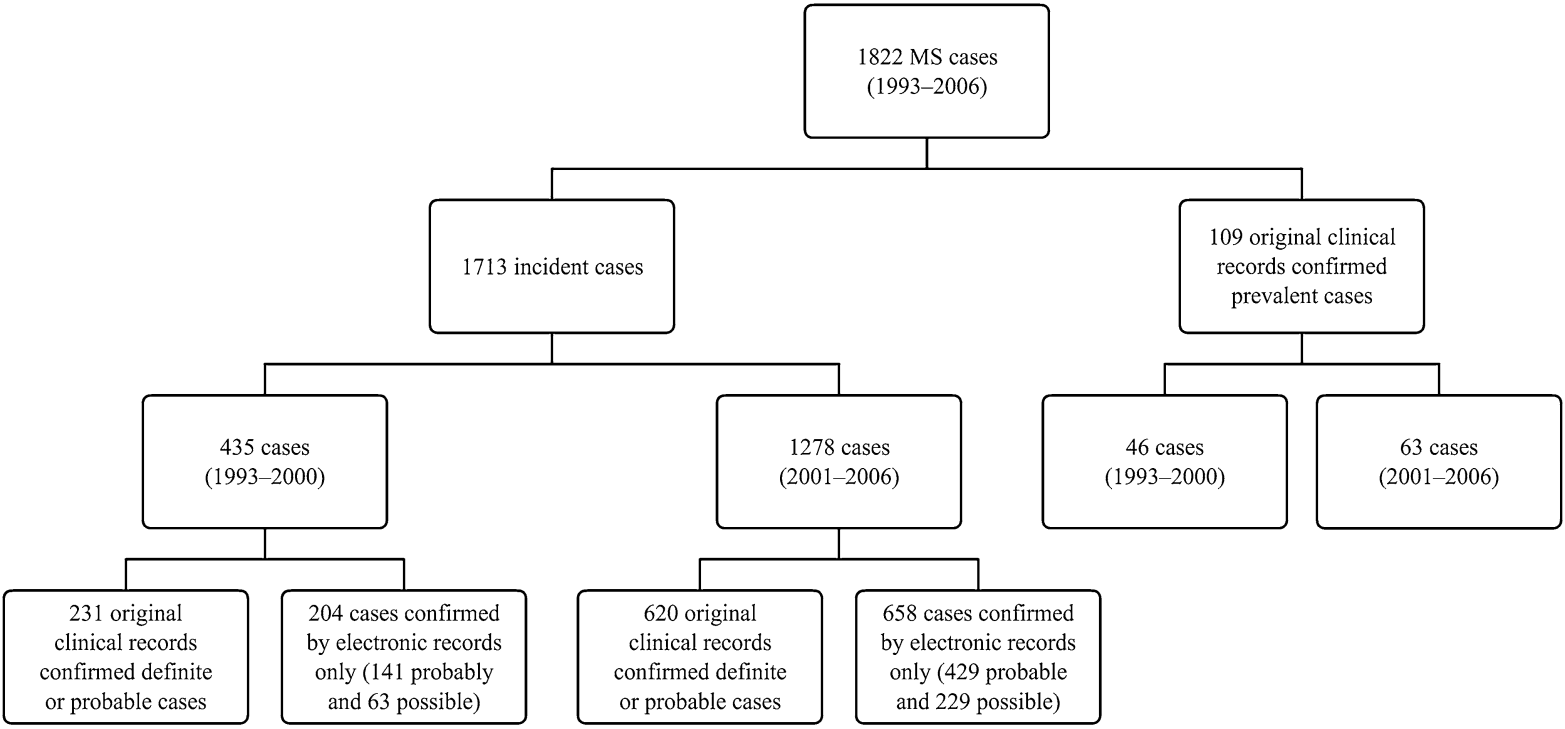
Selection of study sample

We identified 1879 people with a first diagnosis of MS between January 2001 and December 2006. Of these, 998 (53.1 %) had available paper records. These 998 MS cases were reviewed by a neurologist (G.J.F). Of these, 683 (69 %) were considered to have MS, 87 (9 %) as possible MS and the remaining 228 (23 %) as not MS, yielding a positive predictive value of 68 % (95 % CI 65.5–71.3). Out of 881 cases with unavailable paper records, validation based solely on electronic records yielded 429 probable and 229 possible MS cases (658 cases in total). Altogether 1278 incident and 63 prevalent MS cases were identified from 2001 to 2006 (Fig. 1 for MS validation process). In addition, 435 incident and 46 prevalent MS cases identified between 1993 and 2000 who were still present in the current database were retrieved from previous studies [5–10], giving a total of 1822 MS cases (1507 definite or probable and 315 possible) including 1713 incident and 109 prevalent MS cases (Fig. 1). Because reliable information on MS type and symptoms at onset was only available for incident cases, we restricted the study analyses to the 1713 incident MS cases.

### Patient characteristics

Sixty per cent of the 1713 incident MS cases were first diagnosed between ages 30 and 49 (data not shown); mean 42 years, and a majority were female (74 %). Current or former smoking and alcohol abuse were more prevalent among the MS patients who died during follow-up than those who did not die (*p* < 0.01 for all). Among patients whose diagnosis was made based on original clinical records (*n* = 851), 77 % had RRMS, 14 % had PPMS, 7 % had SPMS and 2 % had an unknown subtype; sensory, motor symptoms, and optic neuritis were the most common symptoms recorded at MS diagnosis (Table 1 shows further data on characteristics of the patient population at first MS diagnosis).

MS patients had a high prevalence of several concomitant conditions and MS treatment (Table 2). Infections were the most frequently recorded acute comorbidity on or after first MS diagnosis, occurring in 80 % of patients. Depression, recorded at any time in the database, was the most frequently recorded chronic comorbidity, occurring in 46 % of patients. Prevalence was higher in MS patients who died during follow-up than those who did not die: 25 vs. 7 %, *p* < 0.0001 for pneumonia and influenza; 52 vs. 31 %, *p* < 0.0001 for urinary tract infection; 12 vs. 5 %, *p* = 0.0009 for diabetes; 16 vs. 3.0 %, *p* < 0.0001 for heart disease; and 23 vs. 5 %, *p* < 0.0001 for cancer.

### Risk factors for all-cause mortality

A total of 115 (6.7 %) MS patients died during 13,573 person-years of follow-up. The mean age at first MS diagnosis was higher in patients who died (51.4) than those who did not die during study follow-up (41.1). Mean age at death was 56.9. Current and former smokers at the time of diagnosis had increased risks of death (adjusted HR 2.0, 95 % CI 1.2–3.4; and adjusted HR 2.5, 95 % CI 1.3–4.5, separately), as well as patients with a history of alcohol abuse at the time of diagnosis (adjusted HR 7.6, 95 % CI 3.2–17.7). Among MS cases confirmed by original clinical records, patients with PPMS had a higher risk of death compared with patients with RRMS (adjusted HR 2.2, 95 % CI 0.9–5.5), although it was borderline statistically significant. Compared with patients with sensory symptoms only, patients with other types of symptoms had increased risks of death, particularly patients with multiple symptoms at onset who had a greatly increased risk (adjusted HR 9.0, 95 % CI 2.2–36.3). These effects were based on the 851 MS cases who had paper records and, therefore, details of first symptoms. We only had data on symptoms for 49 MS patients who died. Thus, this effect was based on a small number of cases, never-the-less, the finding was robust and was stronger when covariates were added to the model; see Table 3 for details. Several comorbidities were found to be significant predictors of death; adjusted HRs (95 % CIs) were: 2.7 (1.6–4.5) for pneumonia and influenza, 4.1 (2.7–6.3) for urinary tract infections, 2.2 (1.2–4.2) for heart disease, and 4.9 (2.9–8.0) for cancer. MS treatment was associated with a reduced risk of death in these data (adjusted HR 0.6, 95 % CI 0.4–1.0). See Table 4.

**Table 3:** Risk of all-cause mortality among incident MS patients

Characteristic	Crude HR (95 % CI)	*p* value	Adjusted HR^a^ (95 % CI)	*p* value
Sex				
Male	1.2 (0.8–1.8)	0.4	1.1 (0.7–1.8)	0.7
Female	Reference		Reference	
Smoking status				
Never	Reference		Reference	
Current	2.7 (1.7–4.3)	<0.0001	2.0 (1.2–3.4)	0.006
Former	2.4 (1.4–4.3)	0.002	2.5 (1.3–4.5)	0.004
Unknown	2.4 (1.3–4.3)	0.003	2.5 (1.3–5.0)	0.007
BMI (kg/m^2^)				
<18.5	2.2 (0.8–6.0)	0.1	1.6 (0.5–5.0)	0.4
18.5–24.99	Reference		Reference	
25.0–29.99	0.8 (0.5–1.4)	0.5	0.8 (0.5–1.4)	0.4
≥30	0.9 (0.5–1.7)	0.7	0.9 (0.5–1.9)	0.8
Unknown	1.0 (0.6–1.6)	0.9	0.9 (0.5–1.6)	0.7
Alcohol abuse	7.5 (3.6–15.7)	<0.0001	7.6 (3.2–17.7)	<0.0001
Type of MS^b^				
RRMS	Reference		Reference	
PPMS	1.7 (0.8–3.6)	0.2	2.2 (0.9–5.5)^c^	0.1
SPMS	3.3 (1.5–6.9)	<0.0001	1.7 (0.6–4.6)^c^	0.3
Unknown	3.2 (0.8–12.5)	0.09	7.7 (1.8–33.3)^c^	0.007
Symptoms at onset^b^				
Sensory	Reference		Reference	
Motor	2.7 (1.0–7.0)	0.04	5.8 (1.8–18.1)^e^	0.003
Optic neuritis	1.8 (0.6–5.4)	0.3	4.0 (1.1–14.6)^e^	0.04
Multiple symptoms^d^	3.6 (1.2–10.8)	0.02	9.0 (2.2–36.3)^e^	0.002
Other	2.0 (0.6–6.6)	0.2	5.2 (1.2–21.4)^e^	0.02
Missing	2.9 (0.9–9.2)	0.08	11.3 (2.9–43.4)^e^	0.0004

*BMI* body mass index, *CI* confidence interval, *HR* hazard ratio, *MS* multiple sclerosis, *PPMS* primary progressive MS, *RRMS* relapsing-remitting MS

^a^Estimates of HRs were obtained in a Cox regression model including sex, year of birth, smoking status, body mass index, a history of alcohol use, acute infection, chronic comorbidities, and MS treatment

^b^Among 851 MS cases confirmed by original records (1993–2006)

^c^Estimates of HRs were obtained in a Cox regression model including sex, year of birth, smoking status, body mass index, a history of alcohol use, acute infection, chronic comorbidities, MS treatment, and type of MS

^d^49 MS patients with multiple different symptoms at onset

^e^Estimates of HRs were obtained in a Cox regression model including sex, year of birth, smoking status, body mass index, a history of alcohol use, acute infection, chronic comorbidities, MS treatment, and symptoms at onset

**Table 4:** Comorbidities and MS medications and risk of all-cause mortality among incident MS patients

	Model 1		Model 2	
	Crude HR (95 % CI)	*p* value	Adjusted HR^a^ (95 % CI)	*p* value
Acute infections^b^				
Pneumonia and influenza	4.8 (3.1–7.5)	<0.0001	2.7 (1.6–4.5)	0.0001
Other acute respiratory infection	2.2 (1.5–3.3)	<0.0001	1.3 (0.8–2.0)	0.2
Urinary tract infection	4.8 (3.3–7.1)	<0.0001	4.1 (2.7–6.3)	<0.0001
Other infection	2.6 (1.8–3.8)	<0.0001	1.7 (1.1–2.6)	0.02
Chronic comorbidities^b^				
COPD and asthma	1.4 (0.9–2.2)	0.1	1.0 (0.6–1.6)	0.9
Depression	1.4 (1.0–2.1)	0.06	1.0 (0.7–1.5)	1.0
Diabetes	2.4 (1.3–4.3)	0.003	1.2 (0.6–2.5)	0.6
Hypertension	0.9 (0.5–1.4)	0.6	0.7 (0.4–1.2)	0.2
Heart disease	3.1 (1.8–5.3)	<0.0001	2.2 (1.2–4.2)	0.01
Cancer	5.5 (3.4–8.6)	<0.0001	4.9 (2.9–8.0)	<0.0001
Comedications^b^				
MS treatment^c^	0.6 (0.4–1.0)	0.03	0.6 (0.4–1.0)	0.05

*COPD* chronic obstructive pulmonary disease, *CI* confidence interval, *HR* hazard ratio, *MS* multiple sclerosis

^a^Estimates of HRs were obtained in a Cox regression model including sex, year of birth, smoking status, body mass index, a history of alcohol use, acute infection, chronic comorbidities, and MS treatment

^b^Time-varying variables

^c^Includes disease-modifying medications (interferons, glatiramer acetate, mitoxantrone, and natalizumab), steroids, and symptomatic management drugs (tizanidine, baclofen, amantadine HCL, modafinil, and fampridine)

## Discussion

This large, population-based study of 1713 people with incident MS in UK primary care provides important data on the clinical characteristics of this patient population including prognostic factors for mortality. We observed that MS patients suffer from various conditions, with infections and depression the most common, but we also identified several other important predictors for all-cause mortality among MS patients, including smoking, alcohol abuse, multiple symptoms at first diagnosis, pneumonia and influenza, urinary tract infection, heart disease, and cancer.

Others have similarly found that people with multiple comorbidities had an increased risk of mortality [16], and that cardiovascular disease and respiratory infections were associated with higher mortality in MS patients [17–19]. A history of urinary tract infections was also found to be associated with decreased survival in these data; however, these observations are likely to reflect MS severity rather than a causal association. Sex was not associated with mortality in this study. The effect of sex has been shown to vary greatly across studies, over time, and between countries [16–18].

An important objective of this study was to explore whether risk factors and comorbidities for MS interact with the underlying autoimmune process, or the drugs used to treat it, and the extent that these may influence mortality. As expected, we found that people with MS were at high risk of infections of all kinds possibly in part due to the use of immunosuppressant drug treatment. The strong association between pneumonia and risk of mortality could reflect the high risk of pneumonia infection in the MS population in concert with the high risk of death among those who develop pneumonia. We also found that patients who received MS treatments had a reduced risk of death; however, we cannot separate the effects of the drug from the effects of the comorbidities.

Patients who had infections and chronic comorbidities were often found to die of that underlying disease. Among the 29 MS patients who died and who had a history of pneumonia, the pneumonia was considered to be at least one of the causes of death in 22 (76 %) instances. Note that cause of death was not available for all patients who died and pneumonia may have been the cause of death for additional MS patients. This supports the notion that if patients have MS and develop pneumonia, the risk of death is high and perhaps higher than in the general population. Cancer was the known cause of death for 21 (81 %) of 26 MS patients who ever had a diagnosis of cancer in their record and subsequently died. Among the 18 MS patients who had a history of heart disease and died, 13 (72 %) died of cardiovascular disease. In all, 11 of the 115 MS patients who died had MS as the only listed cause of death.

We found that patients with multiple symptoms at MS onset had an increased risk of death compared with those with only sensory symptoms, among cases whose diagnosis was confirmed from their original clinical records during 1993–2006. In a large Finnish cohort of MS patients, Sumelahti et al. [18] reported that optic neuritis or other sensory symptoms at presentation were associated with favourable survival. To the best of our knowledge, the association with multiple symptoms found in this study is new and should be investigated further.

Patients in this study with PPMS compared to RRMS were at increased risk for mortality (adjusted HR 2.2), although it was borderline significant. There were too few people with SPMS (adjusted HR 1.7) to obtain a reliable risk estimate. Compared with other existing studies [16–19], our follow-up was relatively short which could explain the small number of cases, though, since these forms of MS are more severe the higher risk was expected. The average length of follow-up from first MS diagnosis was 7.7 years in our study. The highest HR, however, was found among people with unknown MS type. First, this result was based on a small number of patients and is thus not stable. Second, we were less likely to obtain original clinical records for patients who had died, so this group of MS patients is not a random sample of all MS patients but may represent patients with more severe types of MS, which could explain the strong association with risk of mortality.

We found that more patients died from cancer than from heart disease in this study. Since most MS patients in this study were female, this finding mainly reflects cause of death in women where CVD is not a major cause until after menopause. It is noteworthy that more men died from CVD than from cancer in this study. Since the mean age of death in this study was around 57, this finding is consistent with the leading causes of deaths in White women of the same age [20]. The limited follow-up time in this study precluded following people until later age when mortality rates are higher. An alternative explanation is that MS patients could have better control of vascular risk factors compared to the general population. This could be the result of the close medical monitoring received by these patients and the fact that, confronted with a chronic and incurable disease, physicians will strive to correct those comorbidities amenable to treatment in an effort to decrease the total morbidity burden of their patients. Finally, it is also possible that the autoimmune process underlying MS, or the immunomodulatory agents used to treat it, or a combination of both, results in a slower progression of the atherosclerotic process that leads to heart disease.

Another relevant finding of the present study is the association between the infectious processes and mortality in MS patients. It is indeed feasible that this is a spurious association, possibly due to confounding generated by un- or incompletely measured factors associated with both infection occurrence and death. One obvious candidate for this inadequately controlled confounding is treatment for MS, for which we did not have complete information. Therapeutic strategies to confront MS involve modulating the immune system and could thus be related to the incidence and prevalence of infections via decreased immunity, and to mortality via either adverse reactions or disease severity. However, the observation that infections represent a common cause of death in other chronic conditions like Parkinson’s disease [21] suggests that the described statistical association between infection and mortality could also represent a true biological phenomenon. One causal structure that could account for the described association involves the relationship between infections and treatment in terms of mediation rather than confounding: MS immunomodulatory therapies predispose MS patients to suffer infections, which in turn cause death.

Strengths of our study include the large sample size and high quality database. In addition, our findings are generalizable to the UK population as a whole because of the representative population-based nature of the CPRD. Additionally, we were able to validate MS diagnoses by accessing original patient clinical records for a large proportion of cases, which, along with linkage to death registry data, enabled additional clinical information to be obtained. Finally, we used age as the time-scale in the Cox proportional regression models instead of time-on-study, the scale used in most previously reported MS studies, to better estimate the effects of predictive factors for death controlling for age. Moreover, because patients may have developed MS many months or years prior to their first MS diagnosis, if we had estimated mortality where we followed patients from the first MS diagnosis, left truncation would have been present in this study. Using age as the time scale accounted for the left truncation issue [22] and is more appropriate in this study.

We were, however, unable to validate the MS diagnosis via original records for all patients; thus, it is likely that some misclassification occurred. Any misclassification would likely have been random, non-differential, and any effects on the HRs would likely have been small and biased towards the null. We were also unable to describe the patient population regarding vitamin D status or ethnicity as this information is not systematically recorded by GPs. While it is thought that vitamin D deficiency is associated with the risk of cancer [23] and cardiovascular diseases [24], the magnitude of these associations is not strong; thus, the absence of information is unlikely to materially impact the results of this study. Recent population-based studies have also reported significant differences in the incidence of MS across ethnic groups. Since the vast majority of people in the UK are Caucasian it is unlikely that the inability to control for ethnicity would materially alter the results. Additionally, we were unable to evaluate the influence of the different MS treatments, particularly interferon beta which has been shown to slow disease progression [25, 26] and reduce all-cause mortality [27, 28] in patients with RRMS. Because in the UK, it is mostly prescribed in secondary care and not always captured in GP records, there were very few MS patients in this study who had records for interferon beta. This may have affected the estimates of all-cause mortality among RRMS patients in our study, yet is unlikely to have affected the HRs associated with the observed predictive factors for all-cause mortality. Finally, we did not have information on socioeconomic status (SES) for patients in this study, so we could not evaluate its effect on mortality. Since all people in the UK are covered by the National Health Service, access to medical care due to SES should not have had a major effect on MS care. However, we still cannot rule out the possibility that our results are confounded by SES.

In conclusion, our population-based study suggests that several factors, some of which are modifiable, act in combination with the autoimmune process responsible for the clinical manifestation of MS. Smoking, alcohol abuse, pneumonia and influenza, urinary tract infections, heart disease and cancer were all predictive factors of reduced survival among this patient group. Further work is warranted to evaluate how these factors compare with those in the general population.

## Electronic supplementary material

Supplementary material 1 (DOCX 17 kb)
