# Supplementary material for: Epidemiology of multiple sclerosis: results from a large observational study in the UK
Source: J Neurol. 2015 Jun 13;262:2033–41. doi: 10.1007/s00415-015-7796-2 (PMC4768220; doi:10.1007/s00415-015-7796-2)
Supplement: Supplementary file 1 — Supplementary material 1 (DOCX 17 kb) [file 415_2015_7796_MOESM1_ESM.docx]

**Epidemiology of Multiple Sclerosis:
Results from a Large Observational Study in the UK**

SS Jick, L Li, GJ Falcone, ZP Vassilev, M-A Wallander

Corresponding author: Susan Jick DSc, Boston Collaborative Drug Surveillance Program, Boston University School of Public Health, 11 Muzzey Street, Lexington, MA 02421

Telephone: 781-862-6660; Fax: 781-862-1680; email: [sjick@bu.edu](mailto:sjick@bu.edu)

**Online Resource 1.** Read codes used to identify potential cases of MS.

F20xxxx, F203.00, F21.00, and 666A.00
